# High-performance Fe(Se,Te) films on chemical CeO_2_-based buffer layers

**DOI:** 10.1038/s41598-022-24044-5

**Published:** 2023-01-11

**Authors:** L. Piperno, A. Vannozzi, A. Augieri, A. Masi, A. Mancini, A. Rufoloni, G. Celentano, V. Braccini, M. Cialone, M. Iebole, N. Manca, A. Martinelli, M. Meinero, M. Putti, A. Meledin

**Affiliations:** 1 ENEA, Frascati Research Centre, Via E. Fermi, 45, 00044 Frascati, Italy; 2 CNR-SPIN, Corso Perrone 24, 18162 Genoa, Italy; 3 Physics Department, University of Genova, Via Dodecaneso 33, 16146 Genoa, Italy; 4 Central Facility for Electron Microscopy, RWTH Aachen University, Ahornstraße 55, 52074, Aachen, Germany; 5 Present Address: Thermo Fisher Scientific, Achtseweg Noord 5, 5651 GG Eindhoven, The Netherlands

**Keywords:** Materials science, Condensed-matter physics

## Abstract

The fabrication of a Fe-based coated conductor (CC) becomes possible when Fe(Se,Te) is grown as an epitaxial film on a metallic oriented substrate. Thanks to the material’s low structural anisotropy, less strict requirements on the template microstructure allow for the design of a simplified CC architecture with respect to the REBCO multi-layered layout. This design, though, still requires a buffer layer to promote the oriented growth of the superconducting film and avoid diffusion from the metallic template. In this work, Fe(Se,Te) films are grown on chemically-deposited, CeO_2_-based buffer layers via pulsed laser deposition, and excellent properties are obtained when a Fe(Se,Te) seed layer is used. Among all the employed characterization techniques, transmission electron microscopy proved essential to determine the actual effect of the seed layer on the final film properties. Also, systematic investigation of the full current transport properties *J*(*θ*, *H*, *T*) is carried out: Fe(Se,Te) samples are obtained with sharp superconducting transitions around 16 K and critical current densities exceeding 1 MA cm^−2^ at 4.2 K in self-field. The in-field and angular behavior of the sample are in line with data from the literature. These results are the demonstration of the feasibility of a Fe-based CC, with all the relative advantages concerning process simplification and cost reduction.

## Introduction

Iron-based superconductors (IBSCs) are a class of materials whose superconducting properties raised great interest in the scientific community since their discovery in 2006^1^. The combination of relatively high critical temperature, *T*_c_, (up to 55 K) with high critical magnetic fields and critical current densities, *J*_c_, in fact, makes them very appealing for practical applications in different sectors^2–7^. Among IBSCs, iron chalcogenides drew significant attention notwithstanding the low critical temperature—about 15 K for bulk Fe(Se,Te)—due to the low toxicity and the low structural anisotropy. In view of large-scale power applications conductors in form of wires or tapes are required. Several works show that Fe(Se,Te) can be successfully obtained in form of tapes^8–14^ by exploiting the same technology developed and commercialized for High Temperature Superconductor (HTS) REBCO materials (REBCO, Rare Earth-Barium-Copper Oxide), the so called coated conductors (CCs). This technology is based on biaxially textured superconducting films on metallic substrates^8,10,15–17^, however their potentiality for applications is strongly limited by costs, ultimately related to the high degree of manufacturing process complexity.

In this scenario, the use of iron-chalcogenides such as Fe(Se,Te) could be decisive. In fact, it could lead to a minimization of the CCs system complexity and cost based on two intrinsic Fe(Se,Te) features: on the one side, larger *J*_c_ tolerance to grain-to-grain misalignment, [from 2° to 4° of REBCO to about 10° for Fe(Se,Te)]; and on the other, lower deposition temperatures (e.g. 200–400 °C, in ultra-high vacuum conditions) with respect to REBCO (> 800 °C, in oxidizing conditions). It follows that the CC template can be extremely simplified, moving from the complex multilayered buffer architecture necessary in REBCO CCs to promote a sharp texture and to protect from substrate ion diffusion, to the 1–2 layers required for Fe(Se,Te)^4,9,10,18^. In this case, deposition on bare metallic substrate led to suppression of superconductivity due to metallic ion contamination, but it was demonstrated that single CeO_2_ buffer layer is sufficient to prevent contamination of Fe(Se,Te) film^8^. The possibility of lowering the number of layers, as well as the simplification of the overall process necessary to obtain the successful growth of high-performance superconductors, is what makes IBSC competitive with REBCO for CCs fabrication.

Among buffer layers, it has been shown that CeO_2_ is one of the most suitable to promote high quality Fe(Se,Te) growth, as it was for REBCO^19–24^. It also proved suitable for Pulsed Laser Deposition (PLD) on Ni-W textured substrates. A significant breakthrough towards low-cost Fe-based coated conductor would be the replacement of PLD-grown buffer layer using chemical solution deposition (CSD) for the growth of the buffer^9,25–31^. Recently, it was reported that Zr-doped CeO_2_ films deposited via the CSD method known as Metal Organic Decomposition (MOD) can act as reliable buffers on YSZ substrates, one of the most adopted substrates for applications, for high quality superconducting Fe(Se,Te) films^9,18^.

However, it appeared clear that the presence of a seed layer is essential to the growth of high-quality superconducting Fe(Se,Te). The seed layer was first proposed by Molatta et al.^32^ who deposited Fe(Se,Te) film at high temperature and high rate prior to Fe(Se,Te) deposition under optimal conditions and obtained increased reproducibility of the superconducting properties of Fe(Se,Te) films. The seed layer also allows for the extension of the temperature window for optimal growth down to 200 °C. Even if the seed layer growth conditions lead to off-stoichiometry Fe(Se,Te) film due to Te loss in the film at high temperature^33^ it provides an ideal template for Fe(Se,Te) because the seed allows for homoepitaxial growth of the superconducting layer^32,34,35^, However, its actual role still has to be clarified.

The aim of this study is the detailed investigation of structural and superconducting properties at high magnetic fields up to 18 T of Fe(Se,Te) films grown by PLD with the seed layer^2,9^ on chemically derived CeO_2_ buffer layers. Such a Fe(Se,Te) film displays a good superconducting performance, such as a *J*_c_^s.f.^ = 1.1 MA cm^−2^ at 4.2 K, and a *T*_c_ = 17.1 K. This work builds up on previously published studies from this group^8,9,18^, elucidating the role of the seed layer on the final film properties and analyzing in detail the transport properties of the superconductor prepared under optimal conditions, whose understanding could be useful in the perspective of Fe(Se,Te) film growth on metallic templates.

## Experimental

### Sample preparation

#### MOD buffer layers

OD Zr-doped CeO_2_ (CZO) precursor solution was prepared dissolving stoichiometric amounts of Ce (III) acetate hydrate (Sigma Aldrich, 99.5%) and 5 mol.% Zr (IV) acetylacetonate (Sigma Aldrich, 98%) in propionic acid (Sigma Aldrich, 99.5%). Rotary evaporation was used to remove water and the excess of solvent until a total metal concentration [Ce] + [Zr] = 0.3 M. The solution was deposited on YSZ single crystal substrate (MaTek) by spin coating at 2000 rpm for 60 s and dried for 5 min at 120 °C in air^9,26^. The pyrolysis thermal treatment was carried out in static air (30 min at 450 °C) and the crystallization treatment in flowing 5% Ar-H_2_ (30 min at 950 °C in 0.5 l/min Ar-H_2_)^26^. Films approximately 20 nm thick are thus obtained.

#### PLD Fe(Se,Te) films

Fe(Se,Te) films were deposited by pulsed laser deposition (PLD) in an ultra-high vacuum chamber (residual gas pressure during deposition of about 10^−8^ mbar during deposition) equipped with a Nd:YAG laser at 1024 nm and using a polycrystalline target with a nominal composition FeSe_0.5_Te_0.5_ synthesized using a two-step method^2^. First, a non-superconducting seed layer of about 100 nm was deposited at 400 °C at high laser repetition rate (10 Hz). Then, the sample was cooled to 200 °C for 100 nm top-layer deposition at 3 Hz. The films were deposited keeping the following parameters: 2 J cm^−2^ laser fluency (2 mm^2^ spot size) and a 5 cm distance between target and sample^2^.

### Sample characterization

Structural characterization was carried out by X-ray diffraction (XRD) using a Rigaku GeigerFlex diffractometer equipped with a Cu-radiation source and a monochromator on the diffracted beam in Bragg–Brentano configuration for both *θ*–2*θ* and *ω*-scans. *ω*-scans were fitted using a pseudo-Voigt curve as in^8,9,18^.

XRD (111) *ϕ*-scan measurements were carried out in a SmartLab Rigaku diffractometer equipped with an Eulerian Cradle, using a Cu K_α1_ radiation. Due to the different crystal phases of CeO_2_ (cubic) and Fe(Se,Te) (PbO-like tetragonal), the χ angle was fixed at 54.7° and 65.5° during the scan, respectively as in^8,9,18^.

AFM measurements were performed on a Park Systems XE-150 atomic force microscope operating in non-contact mode at room temperature. Image analysis was performed using the open source Gwyddion software. Root-mean-square (rms) surface roughness was evaluated on 1 μm^2^ area as in^8,9,18^.

For TEM measurements, cross section lamellas were produced by focused ion beam (FIB, FEI Dual Beam Helios NanoLab). STEM EDX mappings were carried out on a Tecnai Osiris TEM operated at 200 kV, equipped with a ‘Super-X’ EDX detector operated at 200 kV^18^.

The electrical resistance as a function of the temperature was measured in dc in a liquid He cryostat by the four-probe method. The critical temperature *T*_c0_ was evaluated as zero-resistance temperature. The width of the superconducting transition ∆*T*_c_ has been calculated as the FWHM of the pseudoVoigt fit of the d*R*/d*T* curve as in^18^.

Critical current measurements and in-field *R*(*T*) measurements were carried out on strips with defined geometry obtained after patterning with the usual optical photolithographic procedures^18^. The strip widths used for this study are 50 μm. Critical current is defined with the electric field threshold *E*_c_ = 1 μV cm^−1^. Samples were mounted in the maximum Lorentz force configuration in a cryo-free cryostat equipped with an 18 T superconducting magnet^18^.

## Results and discussion

### Buffer layer deposition

**Figure 1:**
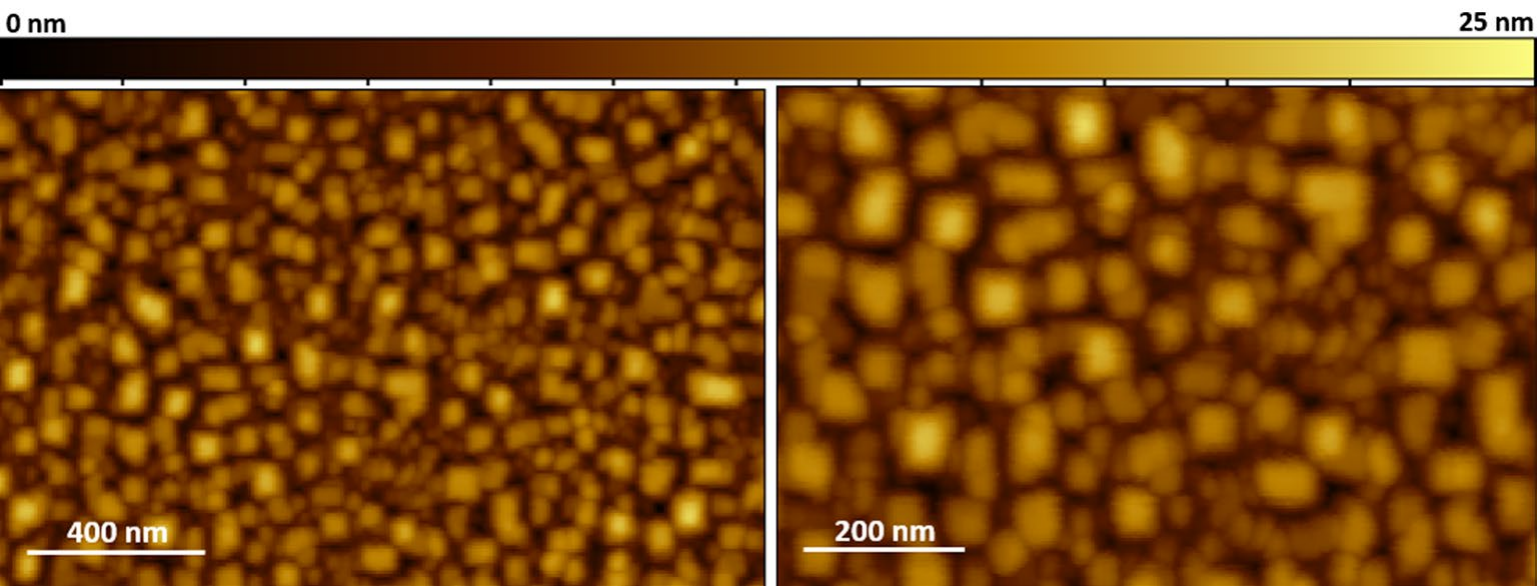
AFM images of a CZO/YSZ film obtained via MOD.

Zr-doped CeO_2_ buffer layers are grown via MOD on YSZ single crystals. Regarding the MOD method, the well-established treatments^9,18,26^ yield high quality results with excellent reproducibility. In Fig. 1 the morphology of a CZO film grown on YSZ via MOD is shown. The film is (00* l*) oriented, with a low value of FWHM_ω−scan_ = 1.2° (data not shown). The microstructure, investigated via AFM, shows small, square-shaped grains are aligned along the < 100 > direction. No pinholes can be seen and the film appears continuous with a value of roughness *R*_*rms*_ = 4.8 nm.

In order to investigate the robustness of the FST_top_ + FST_seed_/CZO/YSZ architecture with respect to the buffer layer roughness, a single seed layer was deposited on a CZO film with a high value of roughness, *R*_*rms*_ = 5.2 nm. AFM analysis after seed layer growth showed a significant reduction in surface roughness, with a *R*_*rms*_ value lowered to 1.5 nm. This implies that the seed layer can compensate for a not-optimal buffer morphology.

To further elucidate this point, a series of dedicated experiments was carried out: three sets of two buffer layers (SET1-3 in Table 1) were selected with three different levels of surface roughness: high, medium and low (specific values are reported in Table 1). These values were selected as the mean *R*_*rms*_ value obtained via the MOD technique (SET2), and the upper/lower limits of the *R*_*rms*_ reproducibility range (respectively SET3 and SET1). Fe(Se,Te) was deposited on every set of buffers, either with or without seed layer. Resistance measurements were then performed on all those films, and for every set of samples the *T*_c_ of the sample with the seed layer is high (approx. 17 K), whereas without seed layer those values are much lower or even unmeasurable. This proves that the seed layer is necessary for the superconductor growth and that it compensate for a significant buffer layer roughness.

**Table 1:** Summary of samples’ relevant parameters.

Sample	Architecture	Buffer R_rms_ (nm)	CZO_(200)_ 2* θ* shift (°)	*a*_CZO_ after FST deposition (%)	FST T_dep_ (°C)	*T*_c_^onset^ (K)
**SET 1**						
FS5221	FST_top_ + FST_seed_/CZO/YSZ	4.3	Δ*θ* = − 0.2	− 1.6	400(seed)/200 (top)	16.9
FS5220	FST_top_/CZO/YSZ	4.3	Δ*θ* = − 0.08	− 0.7	300	–
**SET 2**						
FS5204	FST_top_ + FST_seed_/CZO/YSZ	2.7	Δ*θ* = − 0.14	− 1.1	400(seed)/200 (top)	17.2
FS5205	FST_top_/CZO/YSZ	3.6	Δ*θ* = − 0.11	− 0.8	300	9.5
**SET 3**						
FS5007	FST_top_ + FST_seed_/CZO/YSZ	0.9	Δ*θ* = − 0.26	− 1.9	400(seed)/200 (top)	17.0
FS5006	FST_top_/CZO/YSZ	1.0	Δ*θ* = − 0.16	− 1.2	300	9.1

However, these findings do not clarify the reason why, on a buffer layer with low values of roughness (SET1), the Fe(Se,Te) still requires the presence of the seed layer for optimal growth, with *T*_c_ of only 9.1 K if grown without it. A tentative explanation for this behavior can be given assuming an additional role of the seed layer, namely that of protecting the film from oxygen diffusion from the CZO buffer. In fact, it is well known that CeO_2−*x*_ is a thermodynamically stable oxide in air with oxygen stoichiometry fixed to 2 (i.e., *x* = 0), but it is prone to reduce the oxygen content at high temperature and reduced O_2_ partial pressure^36^. The oxygen loss does not alter the crystalline lattice, retaining the fluorite structure with elongated *a*_0_ lattice parameter. Since the lattice elongation linearly increases with *x*, it derives that changes in *a*_0_ can be interpreted, under proper conditions, as an indication of the oxygen content modification in CeO_2−*x*_^37^. As reported in Table 1, a shift of the CZO (002) peak position to lower values of 2*θ* in the X-ray spectra (due to the lattice parameter elongation) is recognized after heating process for Fe(Se,Te) film depositions performed in vacuum at either 300 °C or 400 °C. Assuming that the low Zr doping level cannot significantly modify CeO_2−*x*_ properties, this indicates that the CZO buffer layer suffers from oxygen loss during Fe(Se,Te) growth. It is therefore reasonable to suppose that the seed layer offers protection from buffer deoxigenation to the Fe(Se,Te) growing film.

From this set of experiments we can conclude that the effect of the seed layer is not only, as previously shown in^9^, to indirectly control the Se/Te ratio by allowing for deposition at low temperatures, but also to compensate for the buffer layer roughness and, possibly, to protect the superconducting layer from oxygen contamination.

Once the optimal conditions are established, films with this architecture can be grown which show properties comparable to Fe(Se,Te) deposited on single crystals.

### Fe(Se,Te) structural characterization

Fe(Se,Te) films were deposited on a CZO buffer layer with using a two-step method^2^. First, a non-superconducting seed layer of about 100 nm was deposited at 400 °C. Then, the sample was cooled to 200 °C for 100 nm top-layer deposition. In previous works by our group it was demonstrated that the optimal temperature for Fe(Se,Te) deposition on CaF_2_ single crystals is 300 °C. This temperature represents a good compromise between the two opposite necessities of favoring epitaxial growth (that requires high temperatures) and avoid Se/Te re-evaporation (that requires low temperature). Indeed, according to Speller's studies^38,39^, there is a large decrease in the tellurium sticking factor above 350 °C, therefore lower deposition temperatures guarantee the maintenance of stoichiometry in the film. To further improve the properties of the films, the two-step method was employed. In fact, using the seed allows you to address the two issues (epitaxy and re-evaporation) separately: the seed layer is deposited at temperatures high enough to promote epitaxial growth and obtain a film with a strong biaxial texture (disregarding the superconducting properties); the top layer is deposited at low temperature using the homoepitaxy mechanism to obtain an optimal stoichiometry and a superconducting top layer. In Fig. S1 the XRD and *R*(*T*) measurements on a seed layer are reported, showing the optimal crystallinity and non-superconducting behavior of the seed layer.

**Figure 2:**
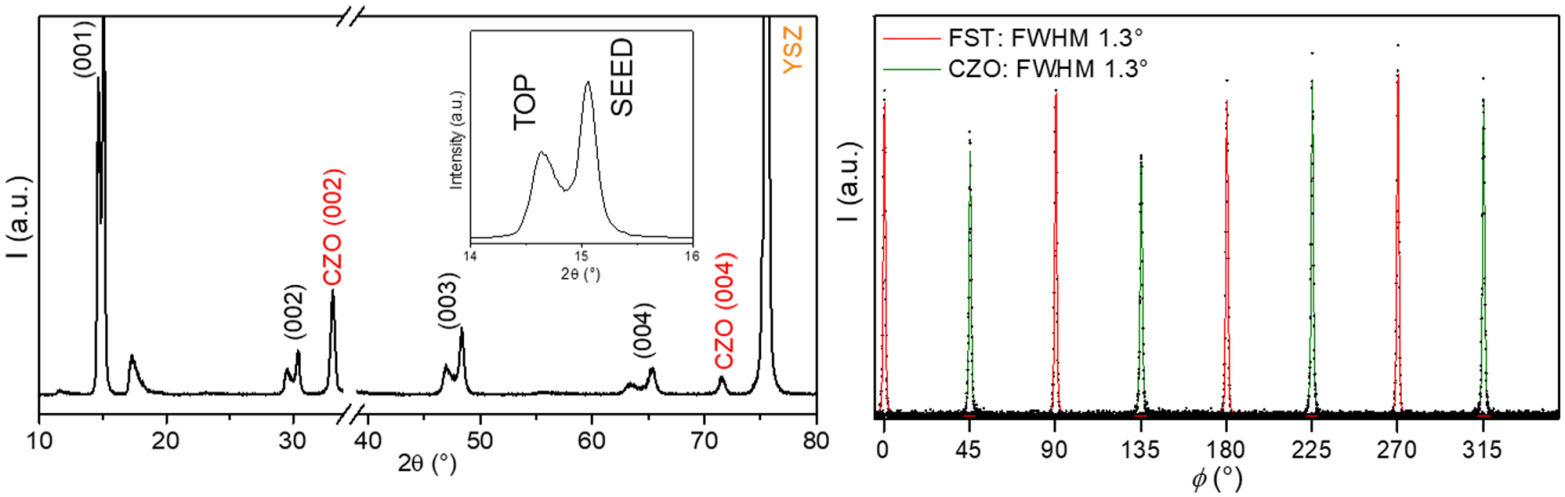
Upper panel: *θ*–2*θ* spectra of a Fe(Se,Te) film (seed layer + top layer) deposited on a MOD CZO buffer. On the left, highlight of the double phase feature of the Fe(Se,Te) signals; Lower panel, : XRD (111) $\phi$-scans of the Fe(Se,Te) film and of the underlying MOD CZO buffer.

In Fig. 2, the XRD *θ*–2*θ* diffraction pattern of a Fe(Se,Te) film deposited on a CZO buffer layer is reported. It shows intense (00* l*) Fe(Se,Te) reflections and signals belonging to both the CZO buffer and the YSZ substrate. Regarding film epitaxy, the Fe(Se,Te) top layer shows optimal out-of-plane orientation, with a (001) ω-scan FWHM = 1.4°, value close to that of the seed layer which shows a slightly sharper distribution (FWHM = 1.2°). The buffer layer, instead has (002) ω-scan FWHM = 1.1° (data not shown). This comparison shows how the excellent orientation is retained from the buffer up to the top layer. In the inset of Fig. 2, the dual-phase nature of the (001) peak is highlighted: this feature points out how the difference in deposition temperatures (400 °C for the seed and 200 °C for the top layer) has an effect on the stoichiometry of the Fe(Se,Te), that causes a significant difference in chemical composition between the seed layer and the top layer. It can be demonstrated that the seed layer peak is at higher 2*θ* values than that of the top layer. As already mentioned in previous works by our group^9,18^, the deposition at 400 °C causes tellurium loss during the process, which results in a Te-poor final film. Such a deviation from the 1:1 stoichiometry is detectable in the decrease of the interplanar distance of the Fe(Se,Te) lattice with respect to bulk Fe(Se,Te), as calculated from the XRD peaks positions. Therefore, a lower angle reflection indicates higher Te content of the film and vice versa. In this case, the calculated lattice parameters are 6.025 Å and 5.874 Å for the top layer and seed layer respectively. *ϕ*-scans of the (111) reflection of the film were also performed and the results are displayed in Fig. 2, lower panel. Both the Fe(Se,Te) film and the buffer layer show sharp peaks, with those belonging to the former rotated by 45° with respect to the latter, thus confirming epitaxial growth of the film on the buffer.

**Figure 3:**
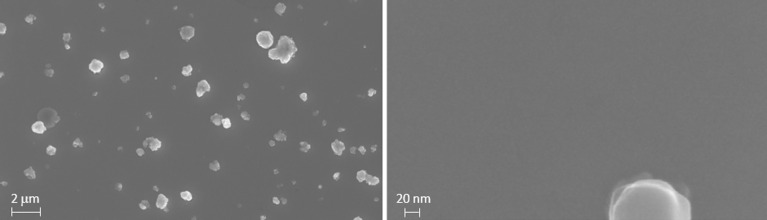
SEM images of a Fe(Se,Te) film (seed layer + top layer) deposited on a MOD CZO buffer.

Microstructural analysis of the sample surface was carried out also via scanning electron microscopy (SEM), and AFM. Figure 3 shows the SEM images of a Fe(Se,Te) film (seed layer + top layer) deposited on a MOD CZO-buffered YSZ at different magnifications. The surface appears very flat and crack-free, with few small outgrowths typical of the PLD derived films. This optimal microstructure is retained also at smaller scales, as can be inferred from the AFM images (not shown). The film is continuous, no pores are visible and the surface roughness is *R*_*rms*_ = 2 nm. It is worth noticing that this value is significantly lower than that of the CZO buffer layer: this again confirms that the rougher morphology of the buffer is compensated for during the Fe(Se,Te) film growth.

**Figure 4:**
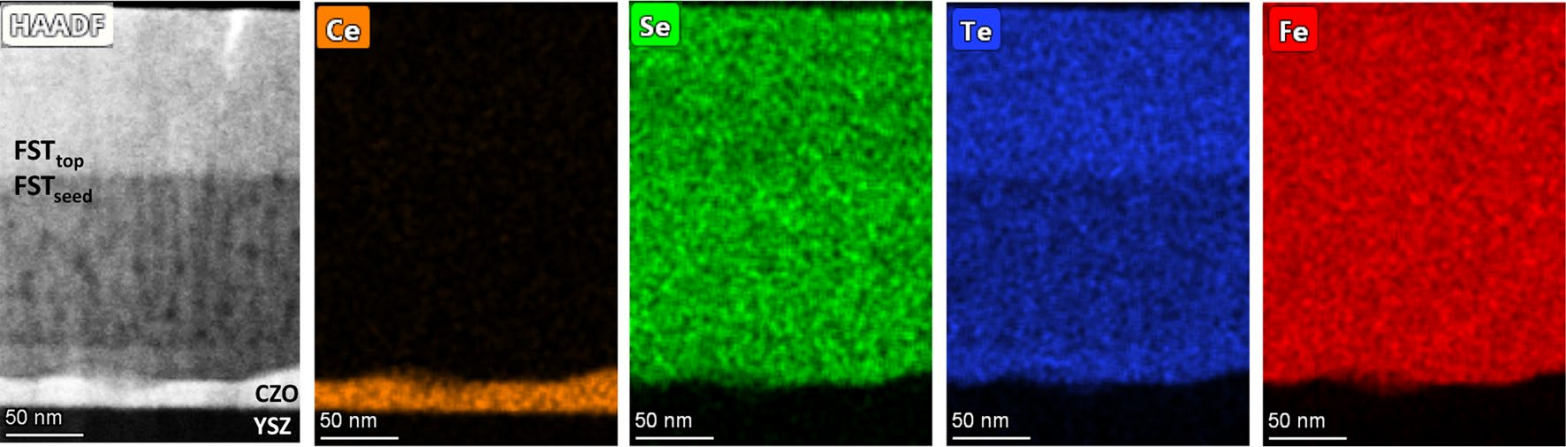
TEM images of a Fe(Se,Te) film (seed layer + top layer) deposited on a MOD CZO buffer. From the left: HAADF image of the film cross section, EDX mapping of Ce, Te, Se and Fe distribution in the same region.

**Figure 5:**
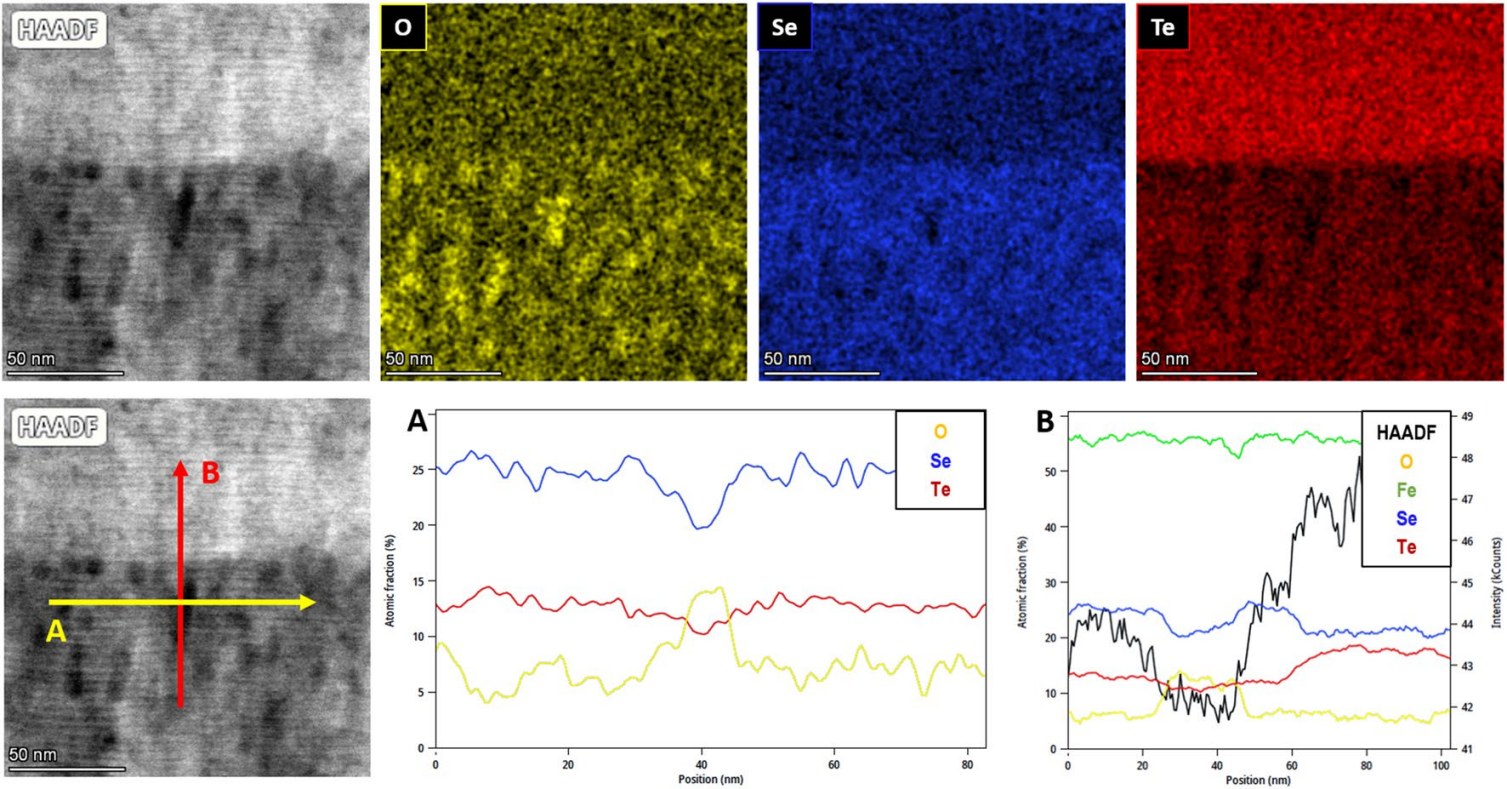
TEM images of the seed/top interface of the Fe(Se,Te) film shown in Fig. 4. From the top-left: HAADF image of the interface, EDX mapping of O, Se, Te in the same region, schematic representation of the lines used to calculate the EDX profiles, EDX profile across a low-intensity region, EDX profile across the interface.

Microstructural characterization is taken further with TEM imaging. In Fig. 4, the TEM images and EDX analyses of a Fe(Se,Te) film (seed layer + top layer) deposited on a CZO buffered YSZ are shown. In the HAADF (High-Annular Angular Dark Field) image we can see (from the bottom): the YSZ substrate, the buffer layer, the Fe(Se,Te) seed layer and the Fe(Se,Te) top layer, both ~ 100 nm thick. Moreover, the interface between seed and top layer is flat and sharp, confirming that the buffer layer roughness is already compensated for during seed layer growth. This supports our previous hypothesis regarding the possibility of loosening the texture requirements of the buffer, thanks to the levelling effect of the seed layer. If elemental analysis is performed on the image, a homogeneous distribution of Fe between the two layers can be seen, whereas different intensities of the Se and Te signals are detected between seed and top layer. More in detail, the Se signal is slightly stronger in the seed than in the top layer, and vice-versa for Te. This different intensity of the Te signal along the *z* direction is in agreement with the XRD data discussed above, confirming that the Fe(Se,Te) top layer is characterized by a lower Se:Te stoichiometric ratio. As regards the buffer layer, the nominal thickness of 20–30 nm is confirmed. It is also worth pointing out the sharpness of the interface between the buffer and the seed layer. No significant diffusion of Ce/Zr atoms is detected in the uppermost layers, further supporting the use of doped CeO_2_ as buffer for Fe(Se,Te). It should be noticed, however, the difference between the microstructures of the seed layer and top layer: in the HAADF image displayed in Fig. 4, darker regions evenly distributed in the seed matrix can be identified; in the top layer, instead, the microstructure is featureless. A close-up of the seed/top layer interface is reported in the HAADF image of Fig. 5, along with the elemental analysis of the region and two EDX profiles, taken along the A and B lines superimposed to the HAADF image. In the EDX maps it is clear how the darker spots visible in the HAADF image correspond to oxygen-rich (and consequently Se/Te-poor) areas. As regards the line scans, Profile A scans a line in the seed layer across one of the darker areas: in the EDX profile the difference in composition can be appreciated, with an enhancement of the O signal in correspondence of the spot, together with the simultaneous decrease of the Se and Te signals. Profile B scans a line across the darker region and across the interface. Again, the profile signals the presence of oxygen inside the darker region, but also the increase of the Te concentration moving from the seed layer to the top layer. The presence of oxygen accumulation in the seed layer is compatible with what previously speculated about the buffer layer’s loss of oxygen during seed layer growth at 400 °C, and its absence in the top layer confirms the of the role of the seed layer as a protective barrier for the superconductor from diffusion of species from the underlying layers. This hypothesis is further supported by the same elemental analysis performed on Fe(Se,Te) films grown without seed layer on CZO buffered YSZ, and Fe(Se,Te) films grown on CaF_2_ both deposited at 300 °C (see Supplementary material). The microstructure of the film grown without seed layer shows the presence of the low contrast regions through the whole film thickness and, again, EDX mapping confirms the presence of oxygen in these areas (Fig. S2). The microstructure of the film grown on CaF_2_, instead, is featureless, without the darker regions (Fig. S3). In this case the EDX map of oxygen does not show relevant features beyond mere diffused contamination. One can wonder why similar effect was never recognized in Fe(Se,Te) films on CeO_2_ buffer layer deposited by PLD techniques which has also been proved to be very effective in preventing Ni contamination from the metallic substrate^8^. The reason of this discrepancy is not fully elucidated yet, but it is believed that the different microstructure of MOD films with respect PLD CeO_2_ (characterized by a large grain size > 100 nm, about 10 times larger than MOD films) can play a major role by promoting oxygen release from the CZO films. This point will be more deeply investigated in the next future.

In conclusion, TEM imaging confirms our previous hypothesis regarding the role of the seed layer: not only it allows for low-temperature deposition of the superconductor providing an excellent template for homo-epitaxial growth, reducing lattice mismatch between buffer and film, but it also offers protection from the CZO-buffer oxygen release and compensates for the buffer layer’s roughness.

### Fe(Se,Te) transport properties

**Figure 6:**
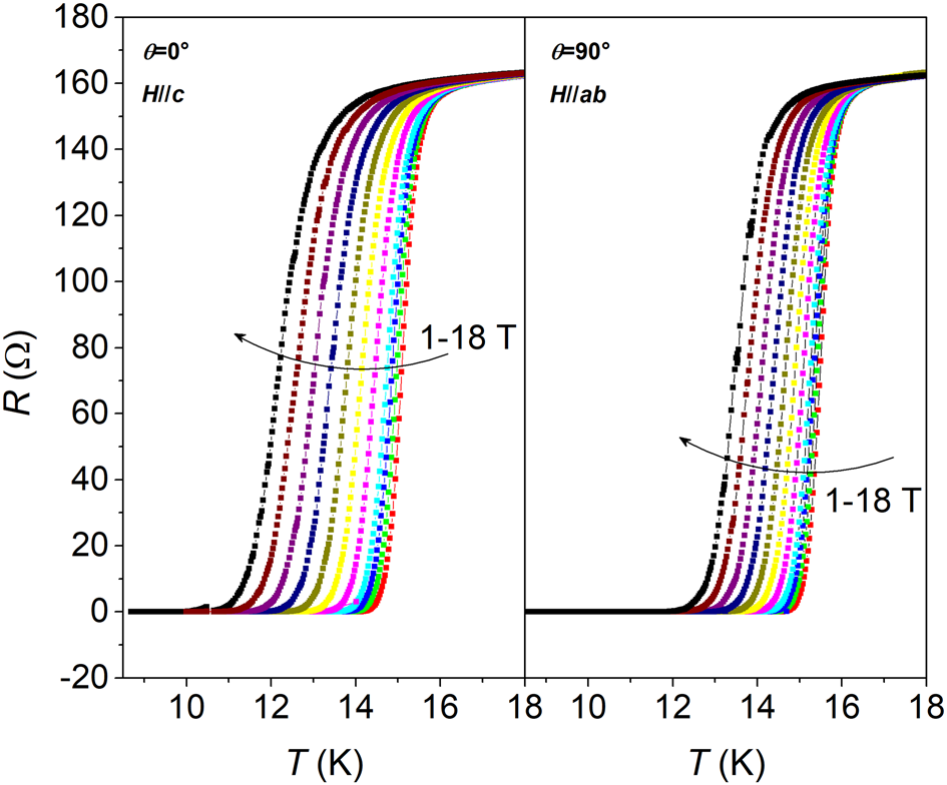
Resistive transitions in applied magnetic fields up to 18 T in a Fe(Se,Te) film (seed layer + top layer) deposited on a MOD CZO-buffered YSZ. Left panel: *H*//*c*, right panel: *H*//*ab.*

The electrical characterization was carried out with the standard four probe method. The *R* versus *T* behavior (see Supplementary material, Fig. S4) is consistent with previous results obtained for this system, with *T*_c_ = 17.1 K and a very sharp transition with Δ*T*_c_ = 0.6 K. These values are very close to those obtained for reference Fe(Se,Te) on CaF_2_ single crystal, which confirms the optimal crystallinity obtained on the buffer layer^9,40^. The sample was then patterned to undergo transport measurements. The in-field *R* versus *T* curves for the patterned sample for *H*//*ab* (*θ* = 90°) and *H*//*c* (*θ* = 0°) are shown in Fig. 6: *T*_c0_ shifts to lower temperatures when the external field is applied (from 15.1 to 11.3 K for *θ* = 0°, from 15.1 to 13 K at *θ* = 90°) and the critical transitions widths slightly increase (from 0.6 to 1.2 K for *θ* = 0°, from 0.5 to 0.9 K at *θ* = 90°). This behavior is commonly related to the vulnerability of the material to thermal fluctuations and considered evidence of thermally activated flux flow^17,41,42^. The dependence from the applied field, is weaker at *θ* = 90° than for *θ* = 0°, which is typically observed in IBS and Fe(Se,Te) in particular^17,42^.

**Figure 7:**
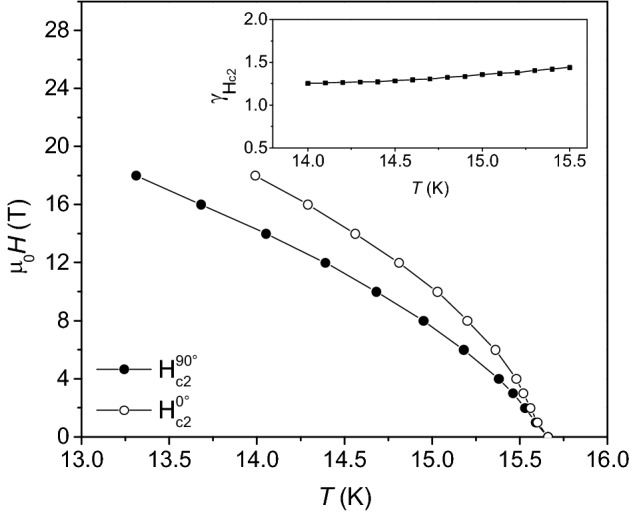
Temperature dependence of H_c2_ derived from R versus T measurements according to the 90% method in a Fe(Se,Te) film (seed layer + top layer) deposited on a MOD CZO-buffered YSZ. Full symbols: *H*//*c*, empty symbols: *H*//*ab.* Inset: anisotropy parameter calculated as γ_*Hc2*_ = $H_{c2}^{//ab} /H_{c2}^{//c}$.

From the in-field *R* versus *T* curves, values of the upper critical fields *H*_c2_ can be estimated. In Fig. 7, the *H*_c2_ values estimated with the criteria of 90% of resistivity at normal state as a function of temperature for both *θ* = 0° and *θ* = 90° are shown. Interpolating the *H*_c2_ values, it is possible to estimate the anisotropy parameter γ_*Hc2*_ = $H_{c2}^{//ab} /H_{c2}^{//c}$ as displayed in Fig. 7 in the inset. The curve exhibits values, in the evaluated temperature range, between approximately 1.2 and 1.5 and an increasing trend with temperature, as shown in the figure. The anisotropy value is similar to what previously reported for Fe(Se,Te)^43,44^ and exhibits a weak dependence on the temperature, often related to the multi-band nature of Fe(Se,Te) superconducting materials. It must be recalled that the crystalline structure is expected to play a role on the critical field and thus on its anisotropy, as suggested observing the effect of epitaxial strain on the electronic states of these materials^45^.

**Figure 8:**
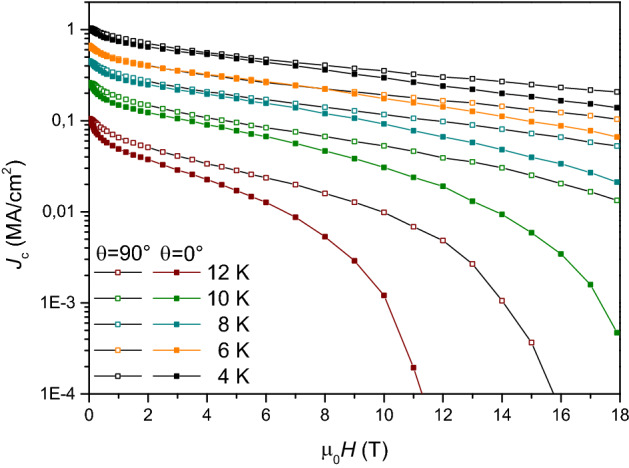
Magnetic field dependence of *J*_c_ at different temperatures in a Fe(Se,Te) film (seed layer + top layer) deposited on a MOD CZO-buffered YSZ. Full symbols: *H*//*c*, empty symbols: *H*//*ab.*

The investigation of the sample is taken forward by measuring transport properties with *H*//*ab* (*θ* = 90°) and *H*//*c* (*θ* = 0°). In Fig. 8, critical current density curves as a function of the magnetic field, *J*_c_(*H*), within 4.2–12 K temperature range for both *θ* = 0° and *θ* = 90°, are shown. At 4.2 K, *θ* = 0° and self-field the sample shows *J*_c_^s.f.^ = 1.1 MA/cm^−2^, and a good in-field behavior at all the investigated temperatures, e. g., at 4.2 K the *J*_c_^18T^ is more that 10% *J*_c_^s.f^; this field dependence is weaker for *θ* = 90° than *θ* = 0° (0.13 MA/cm^−2^ for *θ* = 0°, and 0.21 MA/cm^−2^ for *θ* = 90°). The overall quality of the film is in line with previously published results for Fe(Se,Te) on CeO_2_ buffers^8,9^.

**Figure 9:**
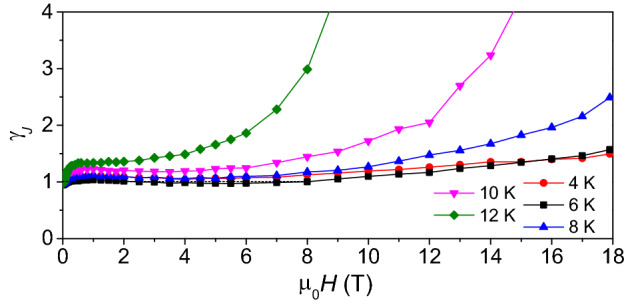
*J*_c_ anisotropy *γ*_*J *_= *J*_c_ (*H*//*ab*)/*J*_c_ (*H*//*c*) as a function of applied field in the 0–18 T field range between 4 and 12 K.

Measurements at *θ* = 90° reveal low anisotropy in the transport properties, the field dependence being slightly weaker for *θ* = 90° than *θ* = 0° (e.g. 0.13 MA/cm^−2^ for *θ* = 0°, and 0.21 MA/cm^−2^ for *θ* = 90° at 4.2 K and 18 T). The critical current anisotropy *γ*_*J*_ = *J*_c_ (*H*//*ab*)/*J*_c_ (*H*//*c*) was calculated for each temperature at different fields and is reported in Fig. 9. Although it is worth recalling that this parameter is not directly related to thermodynamic properties of the material, it is of crucial importance when considering the films in view of the potential application. More in detail, *γ*_J_ is close to 1 in most of the investigated temperature/field range, and it quickly diverges only at higher temperatures and fields, where the irreversibility fields play a major role. At low temperature, the material is substantially isotropic up to 18 T.

**Figure 10:**
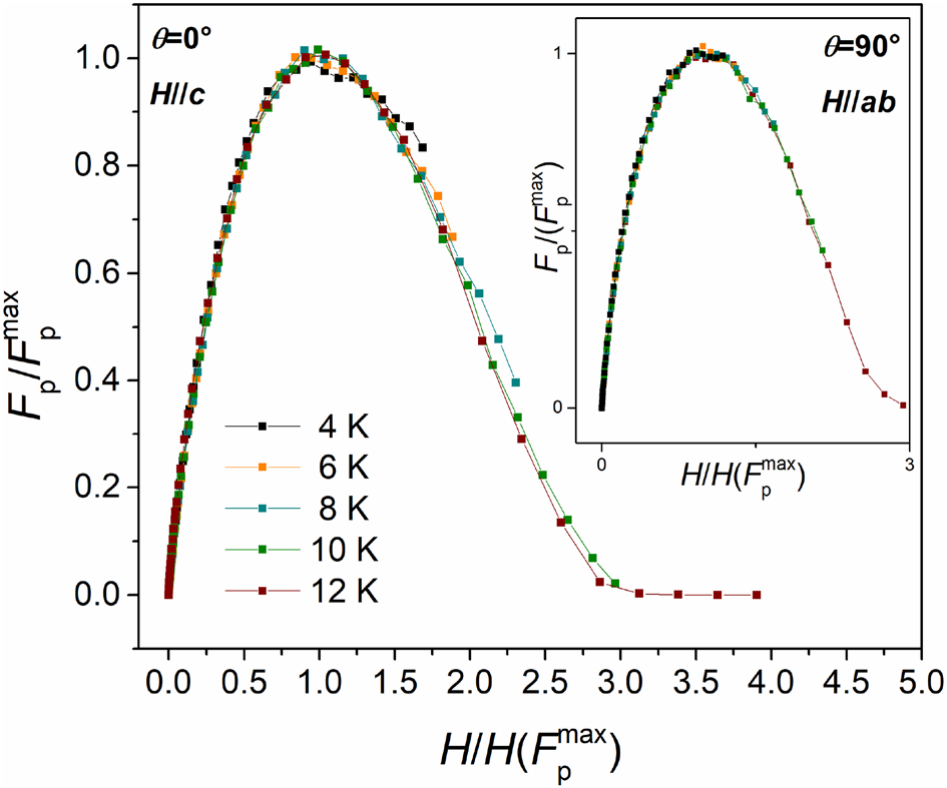
Normalized *F*_p_ curves versus reduced field at *θ* = 0° and *θ* = 90° (inset) showing the overlap of the curves in the whole temperature/field investigated regime.

To analyze in detail the pinning properties of the Fe(Se,Te) film, the scaling behavior of the pinning force densities, *F*_p_ = *J*_c_ $\times$μ_0_*H*, at both *θ* = 0° and *θ* = 90° was investigated. The plot of the normalized pinning forces, $\frac{{F_{{\text{p}}} }}{{F_{p}^{{{\text{max}}}} }}$, versus magnetic field normalized to $H\left( {F_{{\text{p}}}^{{{\text{max}}}} } \right)$, i.e. the field at which the maximum of the pinning force occurs, is shown in Fig. 10. As can be seen, all curves collapse in a single one indicating that a scaling law can be established in the investigated temperature range in either *θ* = 0° (main plot) or *θ* = 90° (inset) conditions. The curves recorded at 12 K were fitted assuming the usual dependence,

$$f_{{\text{p}}} = h^{{\text{p}}} \left( {1 - h} \right)^{{\text{q}}}$$

with: $$f_{{\text{p}}} = \frac{{F_{{\text{p}}} }}{{F_{p}^{{{\text{max}}}} }}$$ and $$h = \frac{H}{{H_{{{\text{irr}}}}^{{{\text{calc}}}} }}$$.

**Figure 11:**
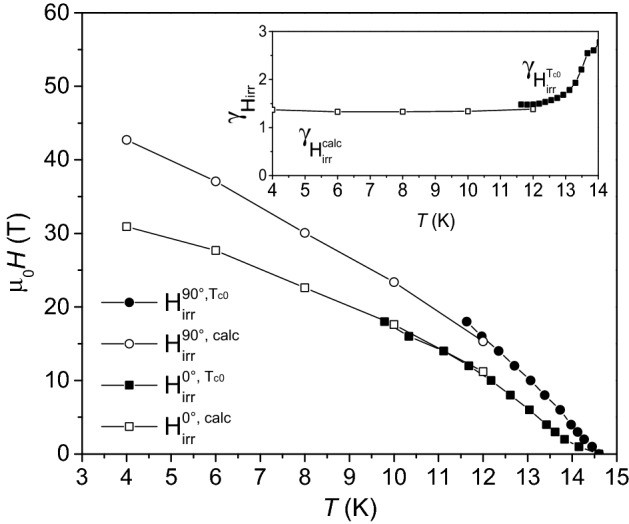
Temperature dependence of H_irr_ derived from R versus T measurements (full symbols) and the pinning force fits (empty symbols) in a Fe(Se,Te) film (seed layer + top layer) deposited on a MOD CZO-buffered YSZ. circles: *H*//*c*, squares: *H*//*ab.* Inset: anisotropy parameter calculated as γ_*Hirr*_ = $H_{irr}^{//ab} /H_{irr}^{//c}$.

With $H_{{{\text{irr}}}}^{{{\text{calc}}}}$, p and q determined by the fitting process. These three parameters are linked to $H\left( {F_{{\text{p}}}^{{{\text{max}}}} } \right)$ by the relationship $H\left( {F_{{\text{p}}}^{{{\text{max}}}} } \right) = \frac{{p H_{{{\text{irr}}}}^{{{\text{calc}}}} }}{p + q}$. By this equation, $H_{{{\text{irr}}}}^{{{\text{calc}}}}$ has been calculated at the remaining (lower) temperatures where the fitting procedure would be less accurate because the experimental data available could not cover the full *h* range (*H*_irr_ > > 18 T approaching 4.2 K). The as obtained values are plotted in Fig. 11 and will be discussed later.

Coming back to the pinning force scaling law, for *H*//*c* the obtained fitting parameters are *p* = 0.8 *q* = 1.6 and *h*_max_ = p/(p + q) = 0.34; for *H*//*ab* instead, *p* = 0.8 *q* = 1.4 and *h*_max_ = 0.35. The results show a significant agreement between the different directions, suggesting that no significant anisotropic contribution is affecting the pinning efficiency, as observed already with the *γ*_J_ graph in Fig. 8. According to the Dew-Hughes model^46^, a set of parameters like *p* = 1 *q* = 1 and *h*_max_ = 0.5 is typical of volume pins, and a set like *p* = 1 *q* = 2 and *h*_max_ = 0.35 is typical of point pins. It is clear how in this case the data do not perfectly fit within any of these two categories. While the value of *p* is typical (close to 1), and the *h*_max_ value points towards point pins, the *q* coefficient is in between the two Dew-Hughes scenarios. More in general, the *q* coefficient affects the behaviour at high reduced fields, and it is an expression of the point-defect pinning efficiency. When the vortex stops behaving as an isolated element that interacts only with the point defects lattice, and starts being affected by the presence of other flux lines, the whole system acquires a collective behaviour and interacts with all the pinning centers. Such a low value of *q* can be related to a high efficiency of the point pins lattice.

To further asses the material anisotropy, an estimation of the irreversibility critical field with respect to temperature for the different orientations was carried out collecting the results from the superconducting transitions (taking into account the zero-resistance critical temperature) and the values obtained from the pinning force fits discussed before. As can be seen in Fig. 11, a good agreement between the two curves can be observed. Using these results, an estimation of the anisotropy γ_*H*_ = $H_{irr}^{90^\circ } /H_{irr}^{0^\circ }$ was performed, and it is reported in the inset. The curve exhibits a relatively constant value of approximately 1.3 for the whole investigated temperature range and an upturn at high temperature. For applications at low temperature, thus, the material shows good superconducting properties and low anisotropy, demonstrating the effectiveness and potential of the proposed architecture.

## Conclusions

This paper reports on the deposition and epitaxial growth of Fe(Se,Te) superconducting films with and without seed layer on Zr-doped CeO_2_ chemical buffer layers and their detailed characterization. The aim of this study is also to investigate and clarify the role of the seed layer on the final film structural and superconducting properties.

The chemical solution deposition method MOD was successfully employed and the quality of the obtained buffers was assessed via evaluation of structural and microstructural parameters. Deposition of the Fe(Se,Te) film/seed layer was performed via PLD. The epitaxial grows was successful and the optimal structure was retained from the buffer to the top layer. Moreover, deeper insight on the role of the seed layer is given: not only it favours chemical matching with the buffer, indirectly controlling the Se/Te ratio by allowing for low temperature deposition, but it also compensates for buffer layer roughness and protects the Fe(Se,Te) film layer from oxygen contamination. Superconducting properties of a Fe(Se,Te) film (seed layer + top layer) deposited on a MOD CZO-buffered YSZ were evaluated via dc measurements and self-field performances resulted in line with data from the literature. Deeper characterization of the film was carried out via angular measurements. The sample shows negligible anisotropy in transport properties visible as a slight difference in *J*_c_ values for *θ* = 0° and *θ* = 90°. The anisotropy parameters *γ*_Hc2_, *γ*_Hirr_ and *γ*_J_ were also evaluated as functions of *T* and *H* and the obtained values are consistent with the low anisotropy of the material, and close to those reported in the literature.

## Supplementary Information

Supplementary Figures.

## Data Availability

The datasets used and/or analysed during the current study available from the corresponding author on reasonable request.
