# Supplementary material for: High-performance Fe(Se,Te) films on chemical CeO2-based buffer layers
Source: Sci Rep. 2023 Jan 11;13:569. doi: 10.1038/s41598-022-24044-5 (PMC9834258; doi:10.1038/s41598-022-24044-5)
Supplement: Supplementary file 1 — Supplementary Figures. [file 41598_2022_24044_MOESM1_ESM.pdf]

:High-performance Fe(Se,Te) films on chemical CeO_2-based buffer layers

L. Piperno[1*], A. Vannozzi[1], A. Augieri[1], A. Masi[1], A. Mancini[1], A. Rufoloni[1], G. Celentano[1], V. Braccini[2], M. Cialone[2], M. Iebole[2,3], N. Manca[2], A. Martinelli[2], M. Meinero[2,3], M. Putti[3], A. Meledin[4*]

1 ENEA, Frascati Research Centre, Via E. Fermi, 45 – 00044 Frascati, Italy
2 CNR-SPIN, Corso Perrone 24, 18162 Genova, Italy
3 Physics Department, University of Genova, Via Dodecaneso 33, 16146 Genova, Italy
4 Central Facility for Electron Microscopy RWTH Aachen University
* currently at: Thermo Fisher Scientific, Achtseweg Noord 5, 5651 GG Eindhoven, The Netherlands

SUPPLEMENTARY MATERIAL

a)

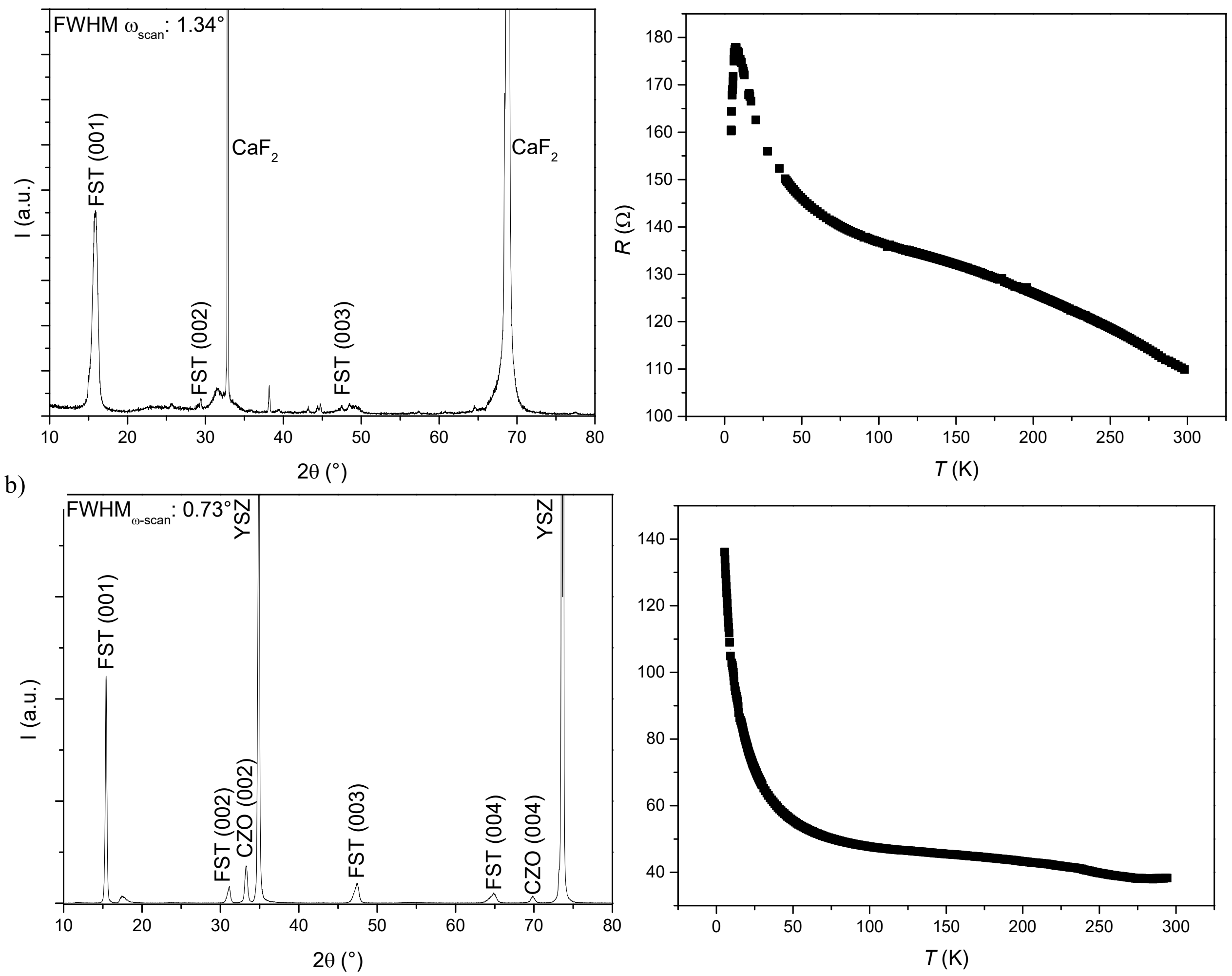

Fig. S1 XRD diffraction and $R(T)$ measurements of a Fe(Se,Te) seed layer grown a) at 400 °C on CaF_2 and b) at 400 °C on CZO/YSZ

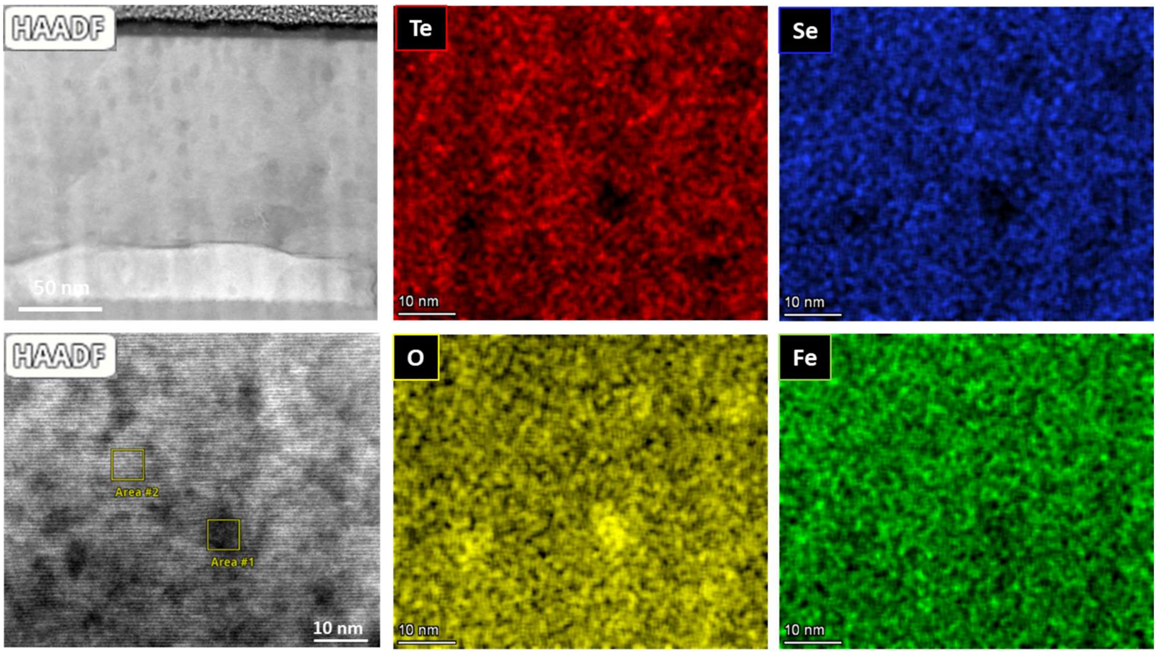

Fig S2 TEM images of a Fe(Se,Te) film (without seed layer) deposited on a MOD CZO buffer. Upper left panel: HAADF image of the film cross section; lower left panel: HAADF image at higher magnification with the related EDX mapping of O, Te, Se and Fe distribution in the same region.

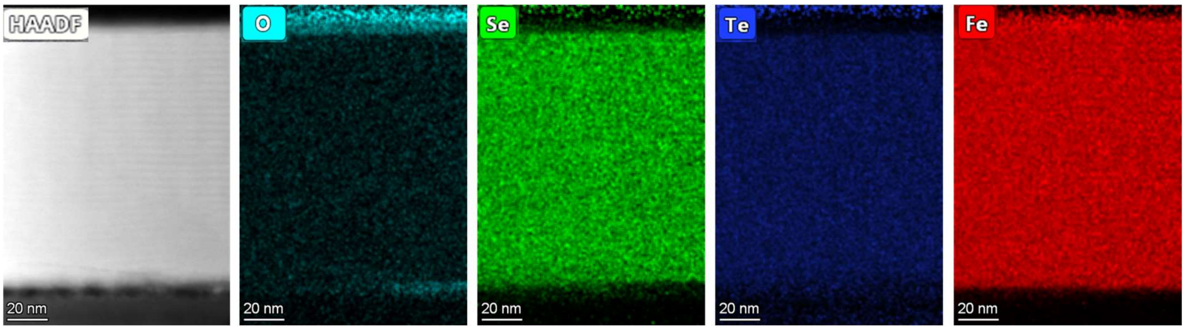

Fig S3 TEM images of a Fe(Se,Te) film (without seed layer) deposited on CaF_2From the left: HAADF image of the film cross section with the related EDX mapping of O, Se, Te and Fe distribution in the same region.

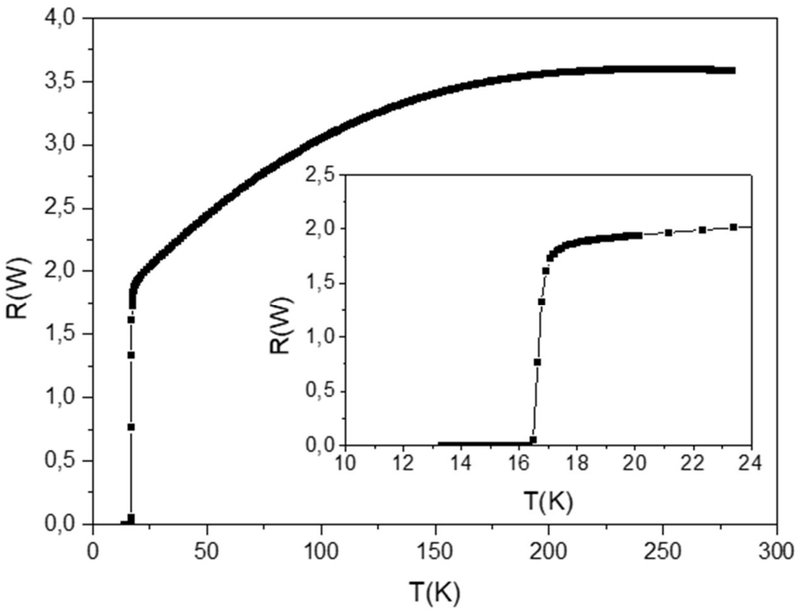

Fig S4 *R* vs *T* of a Fe(Se,Te) film (top layer+seed layer) deposited on a MOD CZO buffer. In the inset, focus on the transition
